# *Coccidioides immitis* and *posadasii*; A review of their biology, genomics, pathogenesis, and host immunity

**DOI:** 10.1080/21505594.2018.1509667

**Published:** 2018-09-04

**Authors:** Theo N. Kirkland, Joshua Fierer

**Affiliations:** a Division of Infectious Diseases, Department of Medicine, University of California San Diego School of Medicine, San Diego, CA, USA; b VA Healthcare San Diego, San Diego, CA, USA

**Keywords:** Fungi, *Coccidioides*, coccidioidomycosis, dimorphism, spherule, genome, transcriptome, immunity, vaccine

## Abstract

*Coccidioides immitis* and *C. posadasii* are two highly pathogenic dimorphic fungal species that are endemic in the arid areas of the new world, including the region from west Texas to southern and central California in the USA that cause coccidioidomycosis (also known as Valley Fever). In highly endemic regions such as southern Arizona, up to 50% of long term residents have been infected. New information about fungal population genetics, ecology, epidemiology, and host-pathogen interactions is becoming available. However, our understanding of some aspects of coccidioidomycosis is still incomplete, including the extent of genetic variability of the fungus, the genes involved in virulence, and how the changes in gene expression during the organism’s dimorphic life cycle are related to the transformation from a free-living mold to a parasitic spherule. Unfortunately, efforts to develop an effective subunit vaccine have not yet been productive, although two potential live fungus vaccines have been developed.

## Introduction

Coccidioidomycosis was first recognized as a fatal disseminated infection 120 years ago, but the fungal etiology was only determined two decades later, and the full clinical spectrum of the disease was not realized for another 40 years after that [1]. In the last decade there have been advances in our understanding of the protective immune response in mice, and modest progress was made toward creating a protective vaccine. Advances in molecular methodology have been used to clarify the taxonomy of the pathogen, the epidemiology of these regionally important, dimorphic, endemic fungal pathogens, and to compare fungal gene expression in its different morphologies. It is still difficult to make precise, targeted mutations in these pathogens, which has limited our ability to identify virulence factors, and to fully understand how the fungus transforms from a mold into the complex and unique parasitic structure (spherule) which is the form it takes within the host. Some of our current knowledge of these issues will be discussed in this selective review.

## Taxonomy

**Figure 1.**
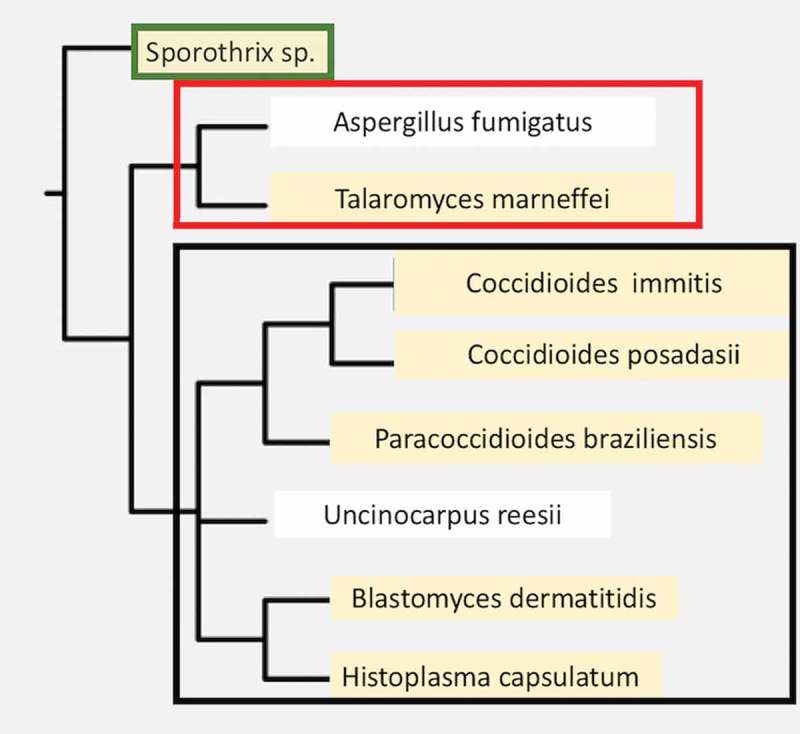
A phylogenetic tree of dimorphic fungi that are human pathogens. A few close relatives that are not dimorphic primary pathogens are shown for comparison (not highlighted in tan). The Orders are shown to the right of the boxed names. Organisms within each Order are boxed together. The phylogenetic data was obtained using the NCBI taxonomy tool and the tree was constructed using Phylip-3.695.

*Coccidioides* species are fungi within Ascomycete division, Eurotiomycetes class, Onygenales order [2,3]. This order includes a variety of dimorphic human pathogens capable of causing invasive disease in immunologically normal hosts, including *Histoplasma capsulatum, Paracoccidioides* spp., and *Blastomyces* spp. The monomorphic mold, *Aspergillus fumigatus*, which is an opportunistic pathogen, is also found in this order. Several non-pathogenic but closely related species are also found within this order (Figure 1). *Coccidioides immitis* and *C. posadasii* are morphologically identical and their predicted proteins are more than 90% homologous [2]. They cannot be distinguished by serologic tests, but the two species can be distinguished by genetic polymorphisms, and some differences in growth characteristics have been reported [4,5]. *C. posadasii* has a larger population size, which is more diverse than *C. immitis* [6]. The biggest difference between the two species is their geographic distribution. *C. immitis* is primarily found in the desert regions of Central and Southern California (including Baja California), while *C. posadasii* is primarily found in desert regions of Nevada, Arizona, New Mexico, West Texas, Mexico, and Central and South America implying that were significant geographic barriers at the time the species diverged from a common ancestor [6]. Some geographic overlap between the two species also occurs in Southern California and Baja California [7]. Both species contain subpopulations that cluster within smaller geographic areas [8].

## Ecology

*Coccidioides* spp. grow in the arid, alkaline desert soil in California, but soil is very complex [9] and the characteristics of contaminated soil may differ between endemic regions [10]. So far, no one set of physical, biological, and chemical characteristic describes all the sites where the organism has been found, and even in one “site” the fungus is distributed unevenly in the soil for unclear reasons. Some evidence exists for the importance of the carcasses of infected small mammals for soil colonization [7], which may explain the spotty recovery from soil samples even in places where small outbreaks have occurred [11], but it is unlikely that infected animals are required for soil colonization [12,13]. In addition to animal carcasses in burrows, it is likely that there is left-over food that the animals brought into the burrows. The genomes of *Coccidioides* reveal an expansion of proteolytic enzymes, which has been interpreted as indirect evidence that the fungus uses proteins (carcasses) as carbon sources in its environmental niches, as it is known that small desert rodents such as kangaroo rats often have the coccidioidal granulomas in their lungs [11].

Recovering the organism from the soil by culture can be difficult [14]. Recovering the organism by mouse inoculation is more sensitive but that technique also has its limitations [15]. *Coccidioides* DNA is found in only a small fraction of soil samples from endemic regions in Baja California and Arizona even by sensitive tests such as PCR [12,15], but a new methodology may increase the sensitivity and specificity of DNA detection [16]. It has been easier to grow *C. immitis* from the soil in the San Joaquin Valley of California in proximity to sites where humans are known to have been infected [7,11,14]. Detecting the organism from the atmosphere is even more difficult than finding it in the soil although recent development of new techniques may improve the sensitivity of detection of aerosolized fungal DNA [17].

## Epidemiology

It is estimated that 30–50% of people in highly endemic areas have been infected, as detected by coccidioidin skin test [18]. These estimates are based on old data as skin tests reagents were unavailable for decades and no large scale epidemiological studies have been done since the recent availability of a spherulin skin test reagent. It is important to remember that the endemic areas include the urban areas of Phoenix, Tucson, Los Angeles, and San Diego, so one does not have to travel to the open desert to contract coccidioidomycosis. Although urbanization probably reduces the risk of infection by decreasing the surface area of exposed contaminated soil, it also increases the numbers of people who can potentially be infected by airborne arthroconidia that are generated by activities that disturb soil, such as construction and earthquakes [19,20]. In addition, windborne spores from an endemic area can infect people and animals many miles from the endemic area [21]. *Coccidioides* are salt tolerant so they may survive in coastal waters, and this could explain how they can infect marine mammals [7,22].

The incidence of reported human infections varies a good bit from year to year, perhaps because of weather patterns. Wet winters seem to correlate with larger numbers of infections in subsequent months [23]. In addition, there has been an overall trend toward an increased number of symptomatic infections over the past 10 years [23,24]. Although the reasons for this increase in incidence are not completely established, in Arizona there are more susceptible people (including older people) moving from non-endemic areas to endemic areas, which is one possible explanation. While the population of the Central Valley of California is not increasing in the same way, the number of cases in suburban Los Angeles County has increased dramatically with urban expansion [18]. In addition, several new prisons were built in the middle of the endemic area in the San Joaquin Valley, resulting in unacceptably high attack rates among the prisoners and guards [25]. Recently a small number of *C. immitis* infections were diagnosed in an arid area in eastern Washington State. Organisms were also isolated from soil samples where the infections were acquired, thousands of miles north of the nearest known endemic areas in California [26]. The soil and human isolates were identical but were genetically distinct from Central and Southern California isolates of *C. immitis*.

There are a number of recent reviews of the clinical manifestations of coccidioidomycosis that contain detailed clinical information [18,27,28]. Pulmonary symptoms are the most common reasons that patients seek medical help, but it is estimated that only 30–50% of infections are symptomatic [18]. Even though most infections are not diagnosed, a large fraction of cases of outpatient pneumonias in Arizona are due to coccidioidomycosis [29]. Nearly all coccidioidal pneumonias are self-limited, even in cases where patients present with extra-pulmonary complications, but in some cases the pneumonia can persist for weeks to months [27,29]. In other cases there may be residual granulomas or thin walled cavities that remain long after the pneumonia is resolved.

Fewer than 5% of immunocompetent patients develop disseminated disease [21,30]. Aside from disease or drug-induced immunosuppression, the risk of disseminated disease is strongly influenced by host factors, such as the third trimester of pregnancy and old age [30,31]. Ethnicity is also a major risk factor for dissemination. In many studies, people who describe themselves as African-Americans are 2–10 times more likely to develop disseminated disease than people of European descent, even when there are no apparent differences in their exposures (such as occurs in prisons and on military bases) [18,25,32]. Filipinos are also more likely to have disseminated infection [33]. The genes and the mechanisms behind ethnic predisposition to dissemination have not been established.

## Genomes

The DNA sequences of several of isolates of *C. immitis* and many *C. posadasii* isolates have been determined [8,34]. There is some hybridization between the two species [6,35]. Sequencing of a large number of clinical isolates in Arizona established that almost all were genetically diverse strains of *C. posadasii* [8]. Groups of isolates from Tucson and Phoenix areas are genetically distinct. A study comparing soil isolates from Phoenix to isolates from patients in the same area found that the environmental isolates were more genetically diverse than the clinical isolates, but there was no evidence for a subset of more pathogenic strains that might explain the increasing incidence of infections in that area [6,34].

The genomes of *Coccidioides* spp. are 28–29 megabases (Mb). At least four chromosomes have been identified by contour-clamped homogeneous electric field gel electrophoresis [36]. The genomes are haploid and no mating has been observed within or between species, although genes coding for mating functions are present [2] and genetic recombination occurs [35]. About 18% of the genome consists of repetitive DNA. *Coccidioides* spp. appear to have a repeat-induced mutation mechanism to control proliferation of transposons [35]. Both DNA and long terminal repeat transposons are found; Gypsy, which is a retrovirus-like transposon that is common in fungi, is the most common type of transposon. Transposons are found more frequently in genomic regions that have few structural genes and are usually found in clusters [37]. The majority of transposons have degenerated and do not have all the domains needed for transposition. There is some evidence that *C. immitis* transposons are preferentially associated with genes coding for protein phosphorylation. In addition, many *C. immitis* genes flanked by some transposon super families are poorly expressed [37]. Of interest, a newly FDA-licensed PCR assay for identifying organisms in clinical specimens and a new modification of that assay that is both sensitive and specific for use on soil samples targets assay targets a copia-like retrotransposon that is present in high copy number in the genome [16].

Gene families coding for phosphotransferase activity, protein kinases, and proteinases, including subtilases and keratinases, are expanded in *Coccidioides* spp. compared to closely related species [3]. This suggests that *Coccidioides* spp. may be specialized for growth on proteins in addition to carbohydrates. There are almost 800 genes that are unique to *Coccidioides* spp. and some are preferentially transcribed in spherules (see below).

## Dimorphism

**Figure 2.**
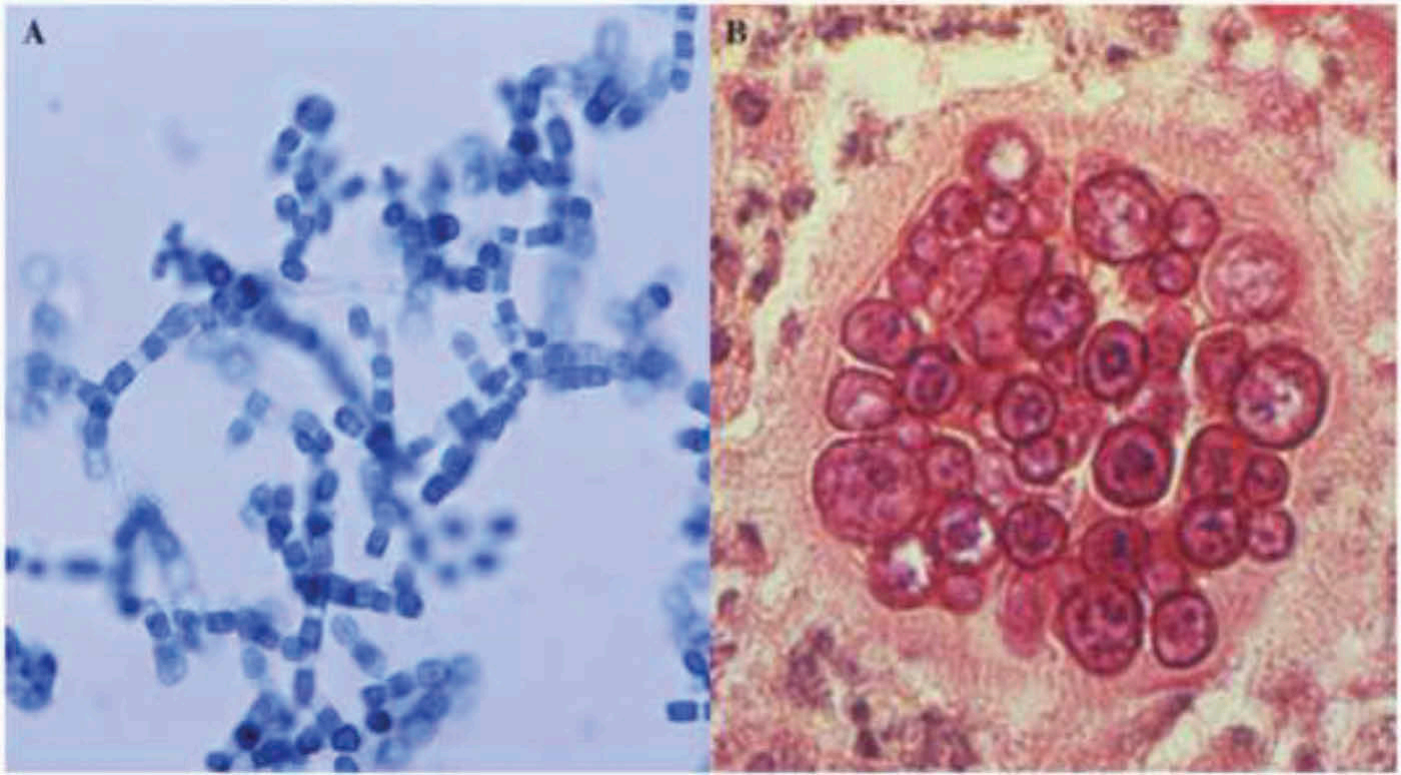
Mycelia containing arthroconidia and a mature spherule with endospores. (a) Mature mycelia grown *in vitro* showing darkly stained arthroconidia alternating with a nucleate thin walled segments within mycelia (lacto-phenol cotton blue stain). (b) A spherule containing endospores in tissue (Periodic Acid Schiff (PAS)). The images were obtained from the CDC (http://phil.cdc.gov/phil/details.asp). This figure was previously published in the Journal of Fungi [70].

All the primary fungal pathogens except *Cryptococcus* spp. share the characteristic of being thermally dimorphic, growing as molds in the soil and differentiating to a yeast or spherule within mammals. *Coccidioides* spp. grow as mycelia (mold) in the soil and form spores known as arthroconidia within the mycelium as they mature (Figure 2). Arthroconidia are released when the contaminated soil is disturbed and each one has the potential to form a new mycelium if it lands in the soil, or a spherule if it infects susceptible animals. The ability to form spherules from arthroconidia is required for pathogenicity. Arthroconidia round up and become immature spherules by isotropic growth. The maturation of spherules involves circumferential swelling of the organism and the synchronous division of nuclei and the cytoplasm to eventually fill the spherule with hundreds of 2 – 4 micron endospores. When a mature spherule ruptures those endospores are released and each one of them has the potential to form another spherule. The differentiation of endospore into mature spherules takes about 4–6 days *in vivo* so the number of spherules can increase very quickly. The transformation from arthroconidia to spherules when grown in a defined medium requires a temperature shift from 25° to 37°, and an increase in atmospheric CO_2_ to 10–14% [38]. It is assumed that similar conditions prompt spherule development *in vivo*, but there is also evidence that contact with neutrophils can stimulate arthroconidia to spherule conversion [39]. In chronic coccidioidal lung cavities, where there are no neutrophils, one can sometimes see reversion to hyphal forms that contain swellings that appear to be abortive attempts to make spherules [40].

Relatively little is known about the change in the transcriptional program that mediates the striking transformation from arthroconidia to spherules. There are only two published studies comparing the transcriptome of mycelia to that of spherules grown in vitro; one studied both *C. immitis* and *C. posadasii* using RNA-seq and the other studied only *C. immitis*, using a whole genome open reading frame microarray [41,42]. The RNA-seq study compared mycelia and day 4 spherules and concentrated on genes that were differentially expressed in both species. 13% of the genes were up-regulated more than two-fold in the spherules of both species, including chitin-associated proteins, β-(1,3) glucan synthase, and the spherule outer wall glycoprotein.

**Figure 3.**
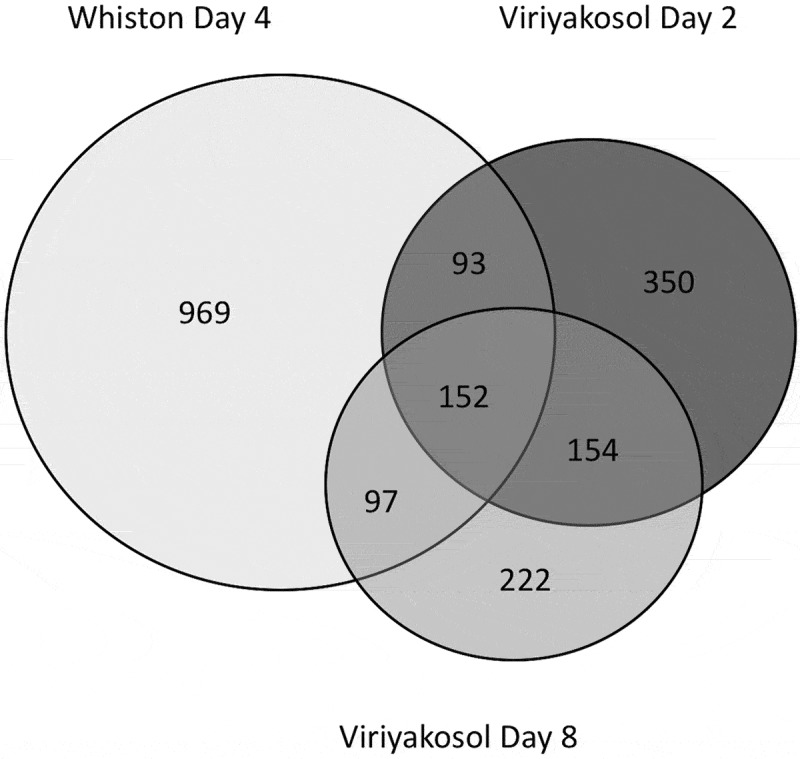
Numbers of genes upregulated in spherules of three different maturities. Venn diagram of *C. immitis* genes up-regulated more than 2-fold compared to mycelial gene expression. (a) The data for day 4 spherules are from [41] and (b) the data for Day 2 and day 8 spherules are from [42].

The microarray study compared *C. immitis* mycelia to day 2 (early in transformation process) and day 8 (endosporulating) spherules. The level of expression of 22% of genes was either up- or down-regulated more than two-fold in day 2 or day 8 spherules compared to mycelia. There were also significant differences between the transcriptomes of day 2 and day 8 spherules (Figure 3). Oxireductases (including extracellular superoxide dismutase that could help protect against neutrophil killing) are up-regulated in day 2 spherules, as are sugar transporters, thioesterase, and amylase. About a third of genes that are up-regulated *in vivo* have no assigned function and 5% are only found in *Coccidioides* spp [42]. One of the most interesting gene families that are down-regulated in *C. immitis* spherules is the protein kinase family; 28 of 184 predicted protein kinase genes were downregulated. Some of the down-regulated protein kinase genes coded for cell cycle, cell wall, and stress response related proteins. The relationship between down-regulation of these genes and spherule formation is not clear.

One of the upregulated genes found in both studies is 4-hydroxphenylpyruvate dioxygenase (4-HPPD, or HpdA). This enzyme is part of a complex of genes involved in tyrosine catabolism. 4-HPPD degrades 4-hydroxyphenylpyruvate homogentisate, which is toxic. Homogentisate is further catabolized or oxidized and polymerized to form pyomelanin [43]. 4-HPPD has been found to be up-regulated in the yeast phase of all dimorphic primary pathogenic fungi [44]. Disruption of the 4-HPPD gene in *Talaromyces marneffei* results in a mutant that cannot grow and differentiate into yeast inside macrophages [45]. The authors of this study believe that this phenotype is unlikely to be directly related to the effect of 4-HPPD on tyrosine metabolism because other mutation in the tyrosine catabolism pathway did not have that phenotype so they suggest that 4-HPPD may have other undiscovered properties.

Despite the differences in experimental design and data analysis, there are 152 genes that were up-regulated in spherules in both studies, regardless of the stage of maturation (Figure 3). Many of these upregulated genes are found in enzymatic pathways of complex carbohydrate metabolism (Table 1). The enrichment of genes in complex carbohydrate pathways seems plausible since extensive remodeling and synthesis of new cell walls must be required for transformation into and growth of spherules and endospores. Twenty-six of the common genes up-regulated in spherules were also up-regulated in the process of *Histoplasma capsulatum* differentiation into yeast [46]. In addition to 4-HPPD, these include several sugar transporters, a sulfite transporter, amylase, keto-reductases, a protein kinase, and a polyketide synthase gene. There have been no published transcriptome studies of *Coccidioides* spherules *in vivo*.

**Table 1.** Some enriched metabolic pathways among the 152 genes up-regulated in day 2, 4 and 8 spherules.

Name	Bkg count^a^	Result count^b^	Fold enrichment	Odds ratio	p- value (Bonferroni)
UDP-sugars interconversion	39	8	12.85	19.88	4.17E-05
L-galactose degradation	36	7	12.18	18.01	4.40E-04
UDP-L-rhamnose biosynthesis	36	7	12.18	18.01	4.40E-04
UDP-*N*-acetyl-&alpha;-D-fucosamine biosynthesis	36	7	12.18	18.01	4.40E-04
lactose degradation II	36	7	12.18	18.01	4.40E-04
L-sorbose degradation	36	7	12.18	18.01	4.40E-04
dTDP-L-olivose biosynthesis	36	7	12.18	18.01	4.40E-04
D-arabinose degradation III	36	7	12.18	18.01	4.40E-04
D-galactose degradation IV	36	7	12.18	18.01	4.40E-04
GDP-6-deoxy-D-*altro*-heptose biosynthesis	42	7	10.44	14.88	1.33E-03
GDP-6-deoxy-D-*manno*-heptose biosynthesis	42	7	10.44	14.88	1.33E-03

Carbohydrate metabolic pathways found in the 152 genes up-regulated in day 2, 4 and 8 spherules [41,42]. a – all genes with that designation found in the genome; b – genes with that designation found in the 152 genes identified.

Proteomics is another approach that can identify expressed genes in spherules and validate so-called hypothetical proteins. There is one published study using this approach that identified proteins in *C. posadasii* mycelia and spherules [47]. They detected 837 proteins (8% of the total predicted from DNA sequence), 88% of which corresponded to the proteins predicted by genome analysis. They also identified 172 novel proteins, most of which could be attributed to differential spicing of the genes. A transposon protein was expressed, indicating that at least one transposon is still transcriptionally active. Studies like this will undoubtedly improve the annotation of the genome.

While this transcriptomic data can identify genes that are preferentially expressed in spherules, they do not tell us what genes are necessary for arthroconidia to differentiate into spherules and what changes in expression are a consequence of the transformation [46]. The most conclusive way to identify the essential genes for spherule formation is to knock them out or to down-regulate their expression with a regulatory RNA and observe the phenotype.

Only a few deletion mutants have been made due to the difficulty of making targeted mutations. Chitinase 2 and 3 genes are expressed at higher levels in spherules than in mycelia, and they were hypothesized to be important for remodeling the cell wall of maturing spherules [48]. A double knockout of those genes resulted in a mutant that does not produce mature spherules with endospores and is avirulent in mice. This mutant was an effective live attenuated vaccine in mice. The CPS1 gene is also important for virulence as deletion of the gene resulted in spherules that grew more slowly than wildtype, could not endosporulate, and the mutant was avirulent even in highly immunocompromised mice [49]. The CPS1 gene product is not well characterized. The gene was chosen as a target because its homologue is associated with pathogenicity in *Cochiobolus heterostrophus*. In mutant spherules, 33 genes were either up or down-regulated compared to wildtype spherules [49]. It is difficult to infer the function of CPS1 from the up- and down-regulated genes. This mutant is also an effective live vaccine in mice.

Deletion of the gene coding for a spherule outer wall glycoprotein also decreased virulence significantly [50]. The outer wall glycoprotein is produced exclusively in spherules and forms the outermost layer that comes in contact with host cells. This proline-rich protein is also an adhesin that is known to bind to laminin.

Enzymes involved in ammonia metabolism are also important for virulence. Infected tissues in mice are quite alkaline and *Coccidioides* synthesizes both a urease enzyme and ureidoglycolate hydrolase, an enzyme that degrades ureidoglycolate to ammonia and glyoxylate (UGH). A urease and UGH double knockout mutant produces less ammonia and is highly attenuated even in genetically susceptible BALB/c mice, suggesting that the ability to alkalinize their environment is important for pathogenicity [51]. However, because the glyoxylate shunt is also a metabolic pathway in fungi for energy generation in the absence of glucose, the *UGH* mutation may have deleterious effects other than reduced ammonia production.

## Immunology

There is a strong correlation between the type of immune response that people make and the prognosis of acute pulmonary coccidioidomycosis. The majority of people have self-limited pulmonary infections, and they develop delayed type hypersensitivity (DTH) as measured by positive skin tests to coccidiodin or spherulin, but they make only low titers of complement fixing (CF) antibodies. In contrast, patients who go on to dissemination of the infection make high titers of CF antibody and do not develop DTH [30,52]. Although this has been known for many decades, there is little or no understanding of what drives the immune response toward a TH1 (DTH, IFNγ) pathway in people who do well. Clearly CD4 T cells are required since patients with low CD4 counts because of untreated AIDS are at high risk for disseminated infections [53] Patients treat with tumor necrosis factor inhibitors are also at higher risk [54]. We also know from rare human genetic mutations that inability to make or respond to IFNɣ is a risk for dissemination of infection [55,56]. A gain of function mutation in Stat1 also predisposes to disseminated coccidioidomycosis and histoplasmosis, but the mechanism(s) behind this increased susceptibility to invasive dimorphic fungi is not clear [57].

The immune response to infection and vaccination have been studied extensively in mice. It is generally accepted that that the innate immune response determines the nature of the adaptive immune response, and that is also true in experimental coccidioidomycosis. DBA/2, a highly resistant mouse strain makes more IL-12p70, IL-23p19, IFNγ, Stat1, and IL-17a and less IL-10 after infection compared to highly susceptible strains, both *in vivo* and *in vitro* [58,59]. In mice, increased IL-10 production results in increased susceptibility, and conversely, IL-10 KO mice are more resistant than the susceptible parental strain, closely resembling DBA/2, the most resistant inbred strain [60]. The only known driver of those differences in mice is Dectin-1, the β glucan receptor, which is expressed on dendritic and other myeloid cells [61]. There is a difference in the structure and function of Dectin-1 in susceptible B6 mice and resistant DBA/2 mice that is based on alternative splicing of the encoding gene, *Clec7a* [59]. That gene is also alternatively spliced in human cells [62,63], but there are no studies on how that differential splicing affects the human response to spherules.

It is likely that Dectin-1 is also at least partially responsible for generating a TH17 immune response as spherules interact strongly with that C type lectin receptor [59]. A mutation of the IL-17 receptor impairs the ability of mice to develop immunity after vaccination by a live avirulent strain of *C. posadasii* [64]. A patient with a STAT-3 mutation, which affect CD4 TH17 differentiation, developed coccidioidal meningitis [65,66]. Since STAT-3 also is involved in many other signaling pathways it is possible that the low levels of IL-17a found in these patients may not be the only explanation for her susceptibility.

The mechanism whereby spherules and/or endospores are killed *in vivo* is also unknown but must depend indirectly on CD4+ T cells as there is an inverse correlation between the total CD4 count and the risk of disseminated coccidioidomycosis in patients with AIDS [67]. The effector molecules that actually kill or inhibit multiplication of the fungus *in vitro* are unknown. People with chronic granulomatous disease are apparently not more susceptible to these pathogens, nor are mice with an orthologous genetic defect [68], so the NADPH oxidase is not needed. There is some uncertainly about the role of iNOS. A broad-spectrum NOS inhibitor increased the susceptibility of DBA/2 mice to *C. immitis* [69]. In contrast, a *Nos2* mutant mouse strain was not more susceptible [70]. However, that mutation was made in C57BL/6 mice, which themselves make very little NO in response this infection [69].

## Vaccine efforts

Vaccine-induced immunity against coccidioidomycosis seems feasible because symptomatic second infections with C*occidioides spp*. are extraordinarily rare (one cannot exclude subclinical infections that boost immunity in highly endemic areas) [71]. Successful vaccination requires a T-cell mediated immune response with both TH1 and TH17 immune responses playing a role in vaccine mediated protection [64]. There is no evidence that an antibody response is protective in humans as there is no association between inherited or acquired immunoglobulin deficiencies and disseminated coccidioidomycosis, but in mouse models there is conflicting evidence about whether B cells are needed for vaccine-induced immunity [72,73]. Ideally, a vaccine would prevent or drastically reduce the incidence of infection, but one that could prevent symptomatic infection and protect those at high-risk for disseminated disease would be considered successful from a clinical standpoint.

Vaccine candidates were all tested initially in genetically susceptible inbred mice, and many recombinant protein vaccines protect them against small but not against large inocula of arthroconidia. It is now feasible to identify and produce recombinant cloned proteins that can be tested as antigens for a vaccine because the genomes of both *C. immitis* and *C. posadasii* are available, and there are enough completly sequenced genomes so that one can choose highly conserved proteins. Unfortunately, there currently is no way to predict which spherule proteins will elicit protective immunity. It was initially believed that highly expressed surface proteins would make the best vaccines, but not all cell-surface proteins are protective (unpublished observations, TNK). Table 2 shows some of the recombinant proteins that have been tested as vaccines [71]. Protection was assessed by decreases in fungal colony counts from quantitative culture of lungs and spleen, and in some cases by survival. The exact criteria varied from one study to another. Single antigens tend to be modestly protective at best [74–76]. The multiple protein/epitope approach seemed to produce the most promising subunit vaccines [77–79]. One dual protein vaccine was tested in cynomolgus macaques and there was a reduction in the burden of disease but the vaccination but did not produce sterilizing immunity [79]. Despite the promising results in mice and monkeys, the development of a recombinant protein vaccine requires important decisions about the what are the best proteins, the amount of those proteins, adjuvant, formulation, and how many doses will be needed to make an effective vaccine. Perhaps most importantly, a pharmaceutical partner willing to manufacture the product is required, given the relatively small population at risk for infection. Careful planning of a clinical trial is also critical. For all these reasons, it is unrealistic to expect a vaccine to be ready for clinical evaluation in the near future.

**Table 2.** Experimental Recombinant Vaccines.

Antigen	Form	Adjuvant	Activity	References
Ag2/PRA^a^	Protein, DNA	Various	Moderately active	[74]
B-glucanosyltransferase	Protein	CpG-ODN^b^	Moderately active	[75]
Calnexin	Protein	Glucan and Adjuplex	Modestly active	[76]
Aspartyl protease	Protein	CpG-ODN	Moderately active	[77]
CSA^c^	Protein	CpG-ODN and MPLA^d^	Modestly active	[78]
Ag2/PRA and CSA fusion protein	Protein	CpG-ODN and MPLA	Highly active	[78]
Phospholipase, α-mannosidase and aspartyl protease	Protein	CpG-ODN	Highly active	[77]

(a) Antigen 2, also known as proline rich antigen(PRA); (b) Cytosine triphosphate deoxynucleotide- guanine triphosphate deoxynucleotide immunostimulatory polymer, (c) *Coccidioides* specific antigen; (d) Monophosphoryl lipid A. This table is adapted from a previous publication in the Journal of Fungi [71].

## Summary

The study of *Coccidioides* spp. has made significant advances over the past decade. Our understanding of ecology and population biology of these organisms is dramatically improved. The availability of genomic sequence of a number of strains has been invaluable for many aspects of research, including creation of several knockout strains that are highly attenuated and provide important knowledge about pathogenesis. The immunology of coccidioidomycosis and *Coccidioides* spp. vaccines is better understood. Unfortunately, there are significant gaps in our knowledge. More information about ecology of this organism is still needed. Understanding of the biology of transformation from mycelium to spherules is limited at best. More information about the human immune response to the infection is also needed. Despite cloning, expression and testing of a number proteins, a subunit vaccine has not been developed. These and other topics provide many challenges for the future.

## Abbreviations

PCR: polymerase chain reaction
INF: interferon
TNF: tumor necrosis factor
IL: interleukin
Stat: signal transducer and activator of transcription
KO: knocked out
THT: helper cell
NOS: nitric oxide synthetase
NADPH: nicotine adenine dinucleotide phosphate oxidase
